# Causal link between hypothyroidism and gastric cancer risk: insights gained through multivariable Mendelian randomization and mediation analysis

**DOI:** 10.3389/fendo.2024.1388608

**Published:** 2024-06-06

**Authors:** Tianyue Zhang, Jie Qiao, Yuanyou Wang, Yinying Zhou, Hammza Jabbar Abdl Sattar Hamoudi, Mhd Alaa Al Hendi, Jun Wang

**Affiliations:** ^1^ Department of Endocrinology, The Second Affiliated Hospital, Zhejiang University School of Medicine, Hangzhou, Zhejiang, China; ^2^ Department of Endocrinology, Qingtian People’s Hospital, Lishui, Zhejiang, China; ^3^ Department of Gastroenterology Surgery, The Second Affiliated Hospital, Zhejiang University School of Medicine, Hangzhou, Zhejiang, China

**Keywords:** gastric cancer, hypothyroidism, Mendelian randomization, genetic variants, causal analysis

## Abstract

**Background:**

Gastric cancer (GC) is the third leading cause of cancer death worldwide, and hypothyroidism has been identified as a potential influencing factor. Despite known associations between hypothyroidism and various cancers, the causal link between hypothyroidism and GC and potential mediators of this relationship remains unclear. This study aimed to clarify these relationships using Mendelian randomization (MR).

**Methods:**

Utilizing genetic variant information from the FinnGen and MRC Integrative Epidemiology Unit open genome-wide association studies (GWAS) databases, we conducted univariable and multivariable MR analyses to explore the causal relationship between hypothyroidism and GC risk. The analysis was adjusted for confounders such as BMI, smoking status, and alcohol intake, and included mediator MR analysis to examine the role of high cholesterol.

**Results:**

We identified a significant inverse association between hypothyroidism and GC risk (OR = 0.93, 95% CI= 0.89–0.98, P = 0.003), with no evidence of reverse causation or pleiotropy. Adjustments for *Helicobacter pylori* infection weakened this association. Mediator analysis highlighted high cholesterol levels, chronic hepatitis B infection, and diabetes/endocrine disease status as significant mediators of the protective effect of hypothyroidism on GC risk.

**Conclusion:**

Our findings suggest that hypothyroidism may confer a protective effect against GC, mediated in part by high cholesterol and other factors. These results underscore the importance of thyroid function and metabolic health in GC risk, offering new insights for preventive strategies and highlighting the need for further research into these complex associations.

## 1 Introduction

Gastric cancer ranks as the fifth most common cancer and the third most common cause of cancer deaths globally, with 1 million new cases and 784,000 deaths annually (1). Despite the declining incidence rates in most nations, there has been an increase in the prevalence of gastric cancer probably due to the aging population. Therefore, preventing and controlling gastric cancer remain crucial. *H pylori* infection is the most extensively documented risk factor for nocardia gastric cancer. Persistent infection of the gastric mucosa results in gradual progression from atrophic gastritis to intestinal metaplasia. Other risk factors include salted food intake, alcohol consumption, genetic background, etc. (2). Despite our understanding of the abovementioned risk factors, our knowledge of the etiology of gastric cancer is incomplete.

Hypothyroidism can cause weight gain, bradycardia, depression, constipation, hyperlipidemia, anemia, menstrual cycle disturbance, and other symptoms (3). However, hypothyroidism may be overlooked due to a lack of attention to this disease. A comprehensive meta-analysis encompassing studies conducted in nine European countries approximated the prevalence of undiagnosed hypothyroidism to be approximately 0.2–5.3%, varied by sex, age, race/ethnicity, and geographic location (4), while subclinical hypothyroidism affects up to 10% of the population, with the highest prevalence among women and elderly individuals (5). Hypothyroidism has been linked to several types of cancer, although its potential causality has not been definitively established. Thyroid hormone, a pleiotropic factor, controls over numerous cellular processes in various cell types, including cancer stem cells. Dysregulation of deiodinase function and thyroid hormone status has been implicated in the development of tumors (6). While immunoblotting studies have demonstrated that changes in thyroid hormone receptor-α protein levels frequently manifest in human gastric cancer and are linked to distant metastasis, the association between hypothyroidism and gastric cancer remains controversial (7).

Furthermore, the substantial impact of thyroid hormones on the regulation of cholesterol metabolism contributes predominantly to the development of hyperlipidemia in individuals with hypothyroidism (8). Previous research has shown that hyperlipidemia is associated with metastasis in patients with gastric cancer (9), while another study reported that total cholesterol level was negatively associated with the risk of gastric cancer in postmenopausal women (10). Consequently, further research is needed to establish the causal relationship between hypothyroidism and gastric cancer.

Mendelian randomization (MR) is a method that utilizes genetic variants as instruments to infer causal relationships between an exposure and an outcome in observational data. This approach overcomes limitations typically encountered in observational studies, such as confounding and reverse causation. The genetic variants used in MR are randomly allocated at conception, which mimics the randomization process of a clinical trial. This random assignment helps to ensure that the genetic variants associated with the exposure are not confounded with other risk factors. MR relies on three core assumptions: the genetic variant is associated with the exposure; it is not associated with confounders of the exposure-outcome relationship; and it influences the outcome only through the exposure, not through other pathways. Despite its strengths, MR is not without limitations, including potential pleiotropy and population stratification biases. However, when appropriately applied and interpreted, MR provides a powerful tool for causal inference in epidemiological research (11, 12). In addition, multiple MR analyses and mediation analyses allow confounding factors to be considered and mediating factors to be discovered (13).

In conclusion, investigating the association between hypothyroidism and gastric cancer has significant importance in the context of global health. The utilization of MR as a research approach is noteworthy due to its capacity to establish causal relationships, thereby providing more reliable and robust evidence. Furthermore, identifying high cholesterol as a mediating factor has significant clinical implications, including connecting metabolic and oncogenic pathways and improving prevention and management strategies through targeted interventions. Therefore, this study aimed to use multivariable and mediator MR methods to explore the causal relationship between hypothyroidism and gastric cancer risk, with a specific focus on high cholesterol as a potential mediating factor.

## 2 Materials and methods

### 2.1 Study design

**Figure 1:**
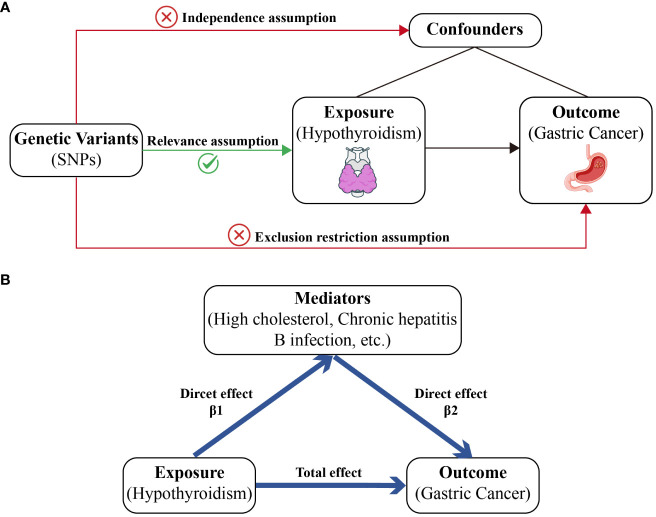
A diagram of our main MR study and mediator MR analysis. This diagram illustrates the potential causal pathway from hypothyroidism to GC, with high cholesterol, IBD, and SRA serving as mediators. **(A)** Main MR analysis. **(B)** Mediator MR analysis.

This study used genetic variations to infer causal relationships between the exposure (hypothyroidism) and the outcome (GC). The three core assumptions underlying MR were as follows: 1) Genetic variants (IVs) should be strongly associated with the exposure (hypothyroidism). 2) These variants should not be associated with confounders of the exposure-outcome relationship, ensuring that the relationship studied is not influenced by external factors. 3) Genetic variants should influence the outcome (GC) only through the exposure (hypothyroidism) and not through other pathways, which is crucial for establishing a causal link (**Figure 1A**).

### 2.2 Data sources

In this MR study, we leveraged data from GWASs to examine the genetic underpinnings of hypothyroidism, its potential mediation by high cholesterol, and the resultant risk of GC. The genetic variants associated with hypothyroidism were sourced from FinnGen (https://www.finngen.fi/en/access_results) (14), a large public-private database that collects and analyses genome and health data from 500,000 Finnish biobank participants. This dataset was labeled as finngen_R9_E4_HYTHY_AI_STRICT and included data from 40,926 patients and 274,069 healthy controls. For the GC dataset, the MRC Integrative Epidemiology Unit (IEU) open GWAS database (https://gwas.mrcieu.ac.uk/) was used (15). The ebi-a-GCST90018849 dataset included a total of 476,116 subjects (1,029 GC patients and 475,087 healthy controls) (16). Additionally, the mediator factor high cholesterol (ebi-a-GCST90029021) (17) was obtained from the IEU open GWAS database. All the datasets were based on European populations (**Supplementary Table 1**).

### 2.3 Selection of genetic instruments

When identifying the genetic instruments used in our study, we meticulously followed a multistep process to ensure the robustness and validity of our analysis. Initially, genetic variants associated with the exposure (hypothyroidism) that reached genome-wide significance (p-value < 5×10^-8^) were considered potential instruments. To mitigate confounding effects and exclude weak IVs, we employed stringent criteria including the exclusion of variants in linkage disequilibrium (LD, r^2^ < 0.001 within a 10,000 kb window) using PLINK software. The F-statistics were calculated for each variant to assess the strength of the instruments, with a threshold of F > 10 to minimize bias due to weak IVs (18). Furthermore, to ensure that the selected SNPs were correlated with the outcome solely via the exposure, we employed the PhenoScanner V2 tool to meticulously assess the phenotypes.

### 2.4 Main and multivariable

In our study, we implemented both univariable and multivariable MR analyses to decipher the causal relationships between hypothyroidism (exposure) and GC (outcome). Univariable MR analysis was conducted to assess the direct impact of the exposure on the outcome, without considering potential mediators or confounders. This analysis utilized the inverse variance weighted (IVW) method (19), a standard approach for MR analyses that combines the weighted median (20), MR Egger (21), simple mode, and weighted mode (22) to estimate the overall causal effect. For the multivariable MR (MVMR) analysis (23), we evaluated the effects of multiple exposures simultaneously, including BMI, smoking status, alcohol intake, and *Helicobacter Pylori*. MVMR utilized genetic variants associated with multiple potential related exposures to estimate the impact of each exposure on a single outcome.

### 2.5 Sensitivity analyses

To ensure the robustness of our causal inferences, we conducted several sensitivity analyses in addition to our primary MR analysis. These analyses aimed to test the validity of our findings against potential biases such as heterogeneity and horizontal pleiotropy. First, Cochran’s Q statistic (19) was assessed through both the IVW and MR Egger regression methods to evaluate heterogeneity. A p-value greater than 0.05 from this statistic suggests an absence of significant heterogeneity among the IVs used. Second, to identify any individual SNP that might disproportionately influence the MR estimate, we performed a leave-one-out analysis. Third, the MR Egger regression method was used to assess the presence of pleiotropic effects among the IVs. Finally, we employed the Steiger test to mitigate the potential for reverse causality.

### 2.6 Mediator MR analysis

Our mediator MR analysis was executed through a bifurcated MR technique to explore whether high cholesterol levels, chronic hepatitis B infection, or other factors mediate the causal pathway from hypothyroidism to GC outcome. Initially, the total effect of hypothyroidism on GC risk was calculated. Second, we determined the causal impact of hypothyroidism on the mediator (β1). Subsequently, the causal influence of the mediator on GC was assessed (β2). To evaluate the significance of the mediating effect (β1 × β2) and its contribution to the overall effect, we applied the delta method (13): the direct effect is equal to the total effect minus the mediated effect (**Figure 1B**).

### 2.7 Statistical analysis

All analyses were performed using TwoSampleMR (version 0.5.8) and MendelianRandomization (version 0.8.0) in R Software 4.3.2 (https://www.R-project.org), along with Storm Statistical Platform (www.medsta.cn/software). Graphics were produced using the ggplot2 package (version 3.4.4) within R. Statistical testing was performed with bidirectional significance tests, where a p-value less than 0.05 was considered to indicate statistical relevance.

## 3 Results

### 3.1 Causal association between hypothyroidism and GC risk

**Figure 2:**
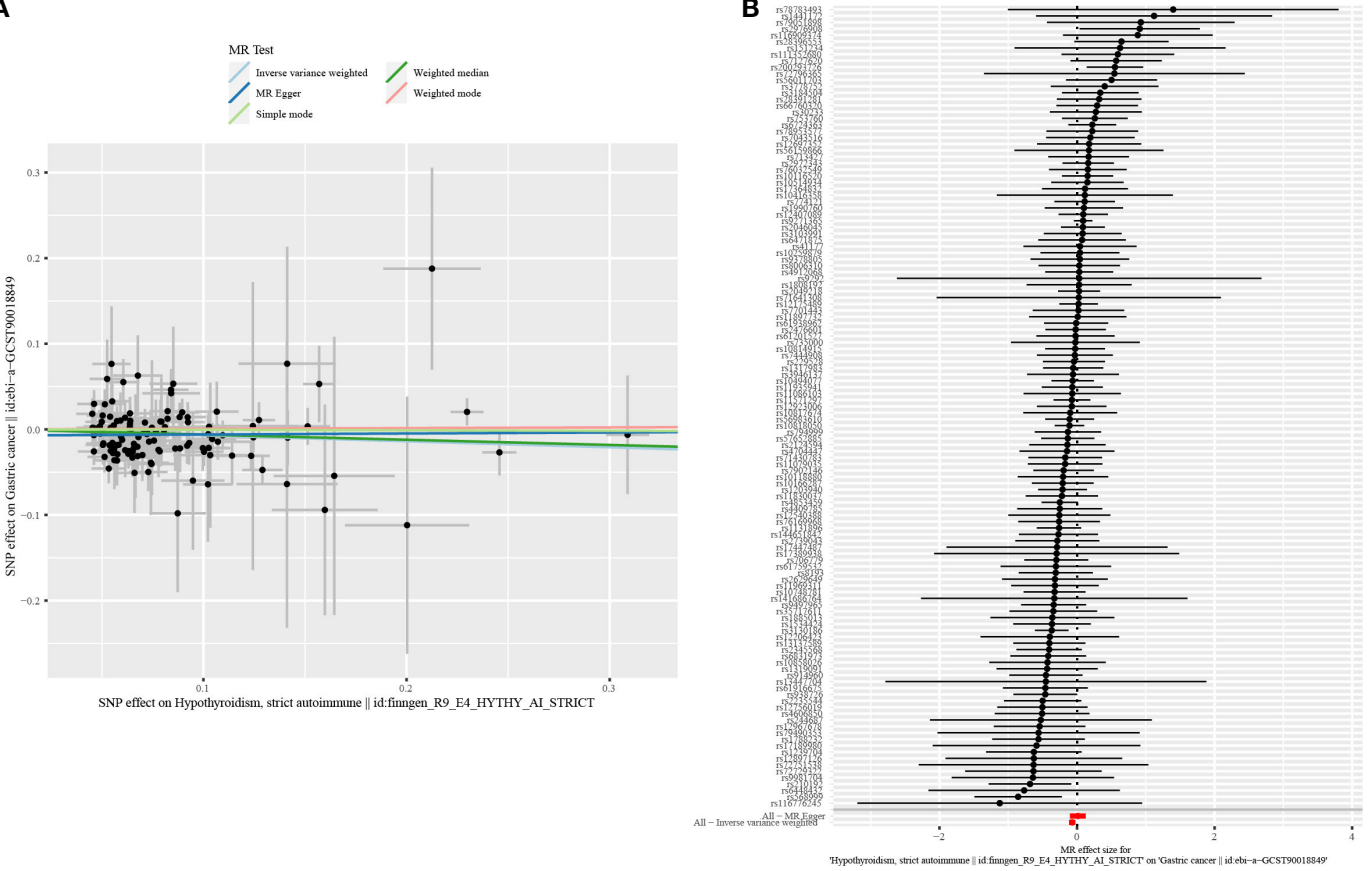
Scatter plot and forest plot for the causal effect of hypothyroidism on GC risk. **(A)** Scatter plot. **(B)** Forest plot.

In this study, according to the criteria of genetic instrument selection, we ultimately identified 125 SNPs significantly associated with hypothyroidism. All the chosen SNPs exhibited F statistic values exceeding 10, which suggested that the instruments employed were of substantial strength (**Supplementary Table 2**). Next, two-sample MR analysis via the IVW method revealed a statistically significant association between a genetic predisposition to hypothyroidism and a decreased risk of GC (odds ratio [OR] = 0.93, 95% confidence interval [CI] = 0.89–0.98, P = 0.003, **Table 1**). Several sensitivity analyses were used to examine and correct for the presence of pleiotropy in causal estimates. No evidence of horizontal pleiotropy was detected (MR-Egger intercept p-value = 0.13). Additionally, Cochran’s Q-test showed no evidence of heterogeneity between these SNPs in terms of causal relationships (**Figure 2**). According to the Steiger test and reverse MR analysis, there was no reverse causality for genetically predicted GC risk on hypothyroidism (IVW method: p-value = 0.1, **Supplementary Table 3**). Thus, there is a causal relationship between the hypothyroidism and risk of GC, suggesting a protective effect of hypothyroidism.

**Table 1:** The main MR results.

Exposure	Outcome	Method	nSNP	Beta	SE	p-value	OR	OR_lci95	OR_uci95
Hypothyroidism	Gastric cancer	Inverse variance weighted	125	-0.06978	0.023421	0.002886	0.932595	0.890752	0.976403
		Weighted median	125	-0.06067	0.03743	0.105016	0.94113	0.874558	1.012769
		MR Egger	125	0.011621	0.05781	0.841008	1.011689	0.903313	1.133067
		Weighted mode	125	0.007567	0.058668	0.897586	1.007595	0.898146	1.130383
		Simple mode	125	-0.00508	0.087917	0.954006	0.994932	0.837445	1.182034

SNP, single nucleotide polymorphism; SE, standard error; OR, odds ratio; CI, confidence interval.

### 3.2 Multivariable analyses adjusting for potential confounders

**Figure 3:**
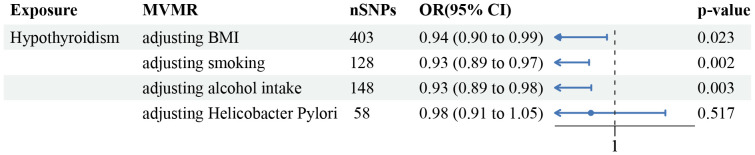
Forest plot of the MVMR analysis. The direct causal effect of hypothyroidism on GC risk was determined by adjusting for traits such as BMI, smoking status, alcohol consumption, and *Helicobacter Pylori* infection status.

In the majority of analyses involving multiple variables, adjustment for three key confounders (BMI, smoking status, and alcohol consumption) did not alter the observed relationship between hypothyroidism and GC risk. However, the link between hypothyroidism and GC risk appeared weaker when the analysis was adjusted for *Helicobacter Pylori* (OR=0.98, 95% CI =0.91–1.05, p-value=0.52, **Figure 3**). Sensitivity analysis revealed an absence of heterogeneity in the IVW and MR Egger tests within the context of multivariable MR assessments. Additionally, there was no horizontal pleiotropy in the MVMR analysis.

### 3.3 Significant mediators of the causal effect of hypothyroidism on GC risk

We performed a two-step mediator MR analysis to investigate the potential mediating roles of several factors, such as high cholesterol levels, chronic hepatitis B infection, and diabetes or endocrine disease in the associations between hypothyroidism and GC risk. Regarding the initiation of hypothyroidism, the IVW method revealed a significant association with a slight increase in the risk of high cholesterol (OR = 1.004, 95% CI = 1.002–1.007, p-value < 0.001) (indicating the intermediary effect β1). Moreover, a causal link between hypothyroidism and a decreased risk of developing GC, was identified via the IVW method (OR = 0.32, 95% CI = 0.18-0.59, p-value < 0.001) (intermediary effect β2). The proportion mediated was estimated to be 6.92% (95% CI = 0.016–0.122, p-value = 0.009), indicating a substantial role of high cholesterol in this causal pathway. Additionally, our findings indicated that chronic hepatitis B infection and diabetes or endocrine disease played roles as mediators; the proportions of mediator effects were 29.13% and 33.98%, respectively (**Table 2**).

**Table 2:** The mediation effect of mediators on the causal effect of hypothyroidism and GC.

Exposure	Mediator	Outcome	Total effect	Direct effect	Mediator effect	Mediator effect proportion%
Hypothyroidism	High cholesterol	Gastric cancer	-0.0698	-0.0650	-0.0048	6.9165
	Chronic hepatitis B infection		-0.0698	-0.0495	-0.0203	29.1293
	Diabetes or endocrine disease		-0.0698	-0.0461	-0.0237	33.9760

## 4 Discussion

To our knowledge, this is the first MR study to investigate whether hypothyroidism is causally associated with gastric cancer. Through a rigorous selection process of instruments and sensitivity analyses to account for potential pleiotropy, the results suggest a noteworthy correlation between genetic susceptibility to hypothyroidism and a reduced likelihood of developing gastric cancer.

While the protective effect of hypothyroidism against GC may seem counterintuitive, emerging evidence suggests that complex interactions between thyroid hormones and various physiological processes are implicated in carcinogenesis. Recent research has shown that hypothyroidism in men may increase the risk of gastric cancer, but the small sample size and study design limit the reliability of these findings (24). Contradicting previous findings, a meta-analysis of 0.5 million patients revealed that hyperthyroidism is linked to a higher risk of gastric cancer, which provided evidence suggesting that hypothyroidism may have a protective effect on the development of gastric cancer (25).

Thyroid hormones play crucial roles in regulating cellular metabolism, growth, and differentiation. These hormones bind to thyroid hormone receptors (TRs), which are widely expressed in various tissues, including the gastrointestinal tract, and modulate gene expression. Dysregulated thyroid hormone signaling has been implicated in various cancers, including breast, thyroid, and gastrointestinal cancers (26). The exact mechanism by which thyroid dysfunction is associated with gastric cancer has not yet been elucidated. There are several potential hypotheses. Evidences suggest that thyroxine may serve as a proliferative factor (27). In addition, decreased stimulation of TRs in the gastrointestinal tract in patients with hypothyroidism may inhibit cellular proliferation and oncogenic signaling pathways, potentially leading to the suppression of tumorigenesis. Primary hypothyroidism will inevitably lead to an increase in TSH. TSH promotes the development of thyroid cancer (28), while a recent study found a negative genetic correlation between TSH levels and breast cancer (29), however, its effect on gastric cancer directly or indirectly is unknown. More research is needed to target certain gene regions downstream (such as key enzymes, metabolites, and signaling, transporters and receptor proteins) to develop preventive strategies and treatments.

Moreover, our study identified several potential mediators that may explain the causal effect of hypothyroidism on GC risk, including high cholesterol levels, chronic hepatitis B infection, and diabetes or endocrine disease. Mediation analysis revealed that high cholesterol, chronic hepatitis B infection, and diabetes or endocrine disease partially mediated the association between hypothyroidism and GC risk.

The role of high cholesterol as a mediator is particularly noteworthy, suggesting a potential link between dyslipidemia and gastric carcinogenesis in the context of hypothyroidism. Many studies have suggested an inverse relationship between cholesterol and gastric cancer, but the mechanism behind this relationship is not well understood. Hypercholesterolemia, which is known to increase oxidative stress and inflammation, is expected to be carcinogenic but this link has not been confirmed for many types of cancer (10). A recent study revealed a strong link between low levels of high-density lipoprotein cholesterol and gastric cancer, regardless of the location of the cancer in the digestive system. The anti-inflammatory and antioxidant properties of high-density lipoprotein cholesterol may contribute to its ability to prevent cancer (30).

Hypothyroidism is associated with dysfunction of glucose absorption and utilization, and research has suggested a potential link between hypothyroidism and both diabetes and hypoglycemia (3, 31). In our MR analysis, diabetes also served as a mediating factor of the effect of hypothyroidism on gastric cancer risk, highlighting the complex interplay among thyroid function, metabolic disorders, and cancer risk. Insulin resistance, and dysregulated hormone signaling pathways may contribute to the development and progression of gastric cancer in individuals with hypothyroidism (32).

Chronic hepatitis B infection also emerged as a significant mediator. A recent MR study demonstrated a causal relationship between hepatitis B infection and gastric cancer (33). However, the specific biological mechanism underlying the association between chronic HBV infection and extrahepatic cancers is unclear. It is hypothesized that HBV protein X, acting as a transcriptional coactivator, may play a pivotal role in the initiation of tumorigenesis by modulating key regulators of apoptosis and interfering with DNA repair pathways and tumor suppressor genes (34). However, further research is needed to investigate the mechanism by which hypothyroidism decreases the risk of gastric cancer through its impact on HBV infection.

There is a close and complex relationship between autoimmune diseases and tumors (35). Systemic lupus erythematosus has been found to be causally associated with a reduced risk of breast cancer (36). The cause of hypothyroidism in our study population was autoimmune thyroiditis, so our results may also suggest a causal association between autoimmune thyroiditis and gastric cancer risk. Further research exploring the specific mechanisms by which autoimmune diseases influence cancer risk remains necessary.

Our study has several strengths including the use of the MR approach, which eliminates some confounders commonly observed in epidemiological studies. Moreover, we used multiple SNPs, that were strongly associated with hypothyroidism. In addition, we further adjusted for multiple confounding factors, indicating that our results are statistically robust. The findings from this study provide compelling evidence for a causal association between hypothyroidism and a decreased risk of gastric cancer. The observed protective effect of hypothyroidism may be mediated by factors such as high cholesterol levels, chronic hepatitis B infection, and diabetes or endocrine disease. However, further research is needed to elucidate the underlying mechanisms and clinical implications of these findings. Besides, due to database limitations, we were unable to discuss other confounding factors such as substance abuse. Nevertheless, understanding the interplay among thyroid function and gastric cancer risk may facilitate the development of novel preventive and therapeutic strategies for gastric cancer.

## 5 Conclusion

Our results suggest that a genetic predisposition to hypothyroidism is causally associated with a decreased risk of GC, with high cholesterol, chronic hepatitis B infection, and diabetes or endocrine disease serving as significant mediators in this pathway. These findings underscore the potential of targeted interventions to modulate mediators as a strategy to mitigate GC risk in individuals with hypothyroidism.

## Data availability statement

The original contributions presented in the study are included in the article/**Supplementary material**. Further inquiries can be directed to the corresponding author/s.

## Ethics statement

Ethical approval was not required for the study involving humans in accordance with the local legislation and institutional requirements. Written informed consent to participate in this study was not required from the participants or the participants’ legal guardians/next of kin in accordance with the national legislation and the institutional requirements.

## Author contributions

TZ: Writing – review & editing, Writing – original draft. JQ: Writing – original draft, Methodology. YW: Writing – original draft, Validation. YZ: Writing – review & editing, Validation, Data curation. HJ: Writing – original draft. MH: Writing – original draft. JW: Writing – review & editing, Visualization, Project administration.

## Supplementary material

Supplementary Table 1 Detailed information of the GWAS datasets included in our study.

Supplementary Table 2 SNPs used as instrumental variables for hypothyroidism.

Supplementary Table 3 Results of reverse causality for genetically predicted GC risk on hypothyroidism.
